# Endometriosis-associated infertility alters the microRNA signatures of cumulus cells with a particularly pronounced effect in oocytes that failed fertilization

**DOI:** 10.1186/s40659-025-00641-2

**Published:** 2025-09-26

**Authors:** Carmen Almiñana, Sofia Makieva, Stefan Bauersachs, Mara D. Saenz-de-Juano, Min Xie, Ana Velasco, Natalia Cervantes, Marianne R. Spalinger, Susanne E. Ulbrich, Brigitte Leeners

**Affiliations:** 1 Department of Reproductive Endocrinology, University Hospital Zurich, Zurich, 8091 Switzerland; 2 Institute of Veterinary Anatomy, Vetsuisse Faculty, University of Zurich, Lindau (ZH), 8315 Switzerland; 3 Animal Physiology, Institute of Agricultural Sciences, ETH Zurich, Zurich, 8092 Switzerland; 4 University of Zurich, Zurich, 8091 Switzerland

**Keywords:** Endometriosis-associated infertility, Oocyte quality, Fertilized oocyte, Cumulus cells, miRNAs.

## Abstract

**Background:**

Endometriosis (E) is multifactorial disease affecting around 10% of women worldwide. The association between E and infertility is clinically well recognized. For E patients to achieve a successful pregnancy, assisted reproductive technologies (ART) are considered as a treatment option. However, the impact of E on oocyte quality, its potential to be fertilized as well as pregnancy rates, is still under debate and with very few molecular clues explaining the clinical data. Alterations in protein-coding RNAs in cumulus cells (CCs), cells surrounding the oocytes and contributing to oocyte maturation, have been reported in E patients. But there is a lack of information regarding microRNAs (miRNAs), which control protein translation. Thus, we aimed: (1) to identify altered miRNA expression in CCs of E patients versus patients without the disease (control, C); and (2) to unveil if in E patients, CCs from fertilized oocytes display a different miRNA profile versus oocytes that failed fertilization. Small RNA-sequencing was performed on CCs from patients undergoing ART.

**Results:**

A total of 85 differentially expressed (DE) miRNAs were identified in E versus C patients (FDR < 0.05). In E patients, 25 DE miRNAs were found between fertilized oocytes and oocytes that failed fertilization, while 13 DE miRNAs in C patients (FDR < 0.05). Comparisons among DE miRNAs highlighted three notable miRNA sets: Set (1) 35 DE miRNAs specific to E; Set (2) 27 DE miRNAs affected by both E and the potential to be fertilized; and Set (3) 6 DE miRNAs characteristic of a competent oocyte successfully fertilized despite the disease. Target gene analysis of DE miRNAs unveiled genes involved in oocyte meiosis, progesterone-mediated oocyte maturation pathway, embryo development, mitochondria and spindle alterations, calcium signaling, and oxidative stress.

**Conclusion:**

This study identified for the first time an altered miRNA signature in CCs of E patients, pointing towards compromised oocyte competence. Besides, in E patients, a characteristic CCs miRNA footprint for oocytes that can be successfully fertilized despite the disease has been revealed. The study charts new territory for non-invasive diagnosis and personalized treatments based on miRNAs to improve oocyte competence in E patients under ART treatments.

**Supplementary Information:**

The online version contains supplementary material available at 10.1186/s40659-025-00641-2.

## Background

Endometriosis (E) is a chronic disease affecting around 10% of women worldwide (190 million, WHO, 2023). It is characterized by the presence of endometrium-like tissue outside the uterus leading to chronic inflammation [1]. Although the cause of the disease remains uncertain, genetic, hormonal, neurological, and immunological factors are all implicated in the mechanisms contributing to the development of symptoms [2]. Thus, E has been defined as a multifactorial disease affecting pelvic anatomy and causing pain, fatigue, alterations in immune and endocrine functions, and infertility. Regarding infertility, impaired folliculogenesis, reduced oocyte and embryo quality, impairment of gamete/embryo transport, as well as dysregulation of functions involved in endometrial receptivity have been described [3]. Because pelvic pain and/or infertility are also associated with other conditions, there is a long delay from the onset of symptoms until definitive diagnosis (7–8 years), which requires surgery [4]. To date there is no definitive cure for E and current E treatments include surgical removal of lesions and drugs that suppress ovarian hormone production which leads to unwanted side effects [2]. Patients surveys intensively demand non-surgical treatments that relief pain and are also fertility-sparing [5].

Since the association between E and infertility is well-known, with 30–50% of women diagnosed with E being infertile and 25–50% of infertile women being diagnosed with E [1, 6], in the recent years, there has been a rise in the number of studies searching for underlying mechanisms in E-associated infertility (reviewed in: [3, 7, 8]. Most of these studies have focused on examining the molecular and functional abnormalities of the endometrium, while the impact of E on the quality of the oocyte itself and associated cumulus cells (CCs) as well as on the potential to be fertilized is still under debate.

CCs are a group of closely associated granulosa cells surrounding the oocytes and supporting their growth and maturation [9]. The maturation of the oocyte is a complex process that relies on the oocyte itself and the communication between the oocyte and the surrounding CCs [10]. CCs contribute to successful fertilization [9] and also protect the oocyte from harmful systemic diseases [11]. For example, diseases such as E, polycystic ovary syndrome (PCOS) or also obesity, diabetes, and environmental toxins can alter hormone, cytokine, chemokine, and reactive oxygen species (ROS) profiles systemically and also locally in the milieu surrounding the oocyte [11] and the CCs can buffer the harmful impact on the oocyte. Under these pathological conditions, the CCs can also be affected, which may alter steroidogenesis and compromise oocyte development and competence [11]. Thus, CCs are critical determinants for oocyte health [12]. As CCs are also exposed to high levels of oocyte-derived regulatory factors that control their functions [13], they have been suggested as non-invasive tools to assess oocyte quality [14, 15]. Gene expression studies of human CCs have identified candidate genes associated to oocyte maturation [16], embryo competence [17], pregnancy and live-birth outcomes [18], but without clear reliable markers of a healthy, mature and fertilizable oocyte [19]. Similarly, messenger RNA alterations in CCs from E patients have been reported, increasing our understanding of the impact of E, but again without clear diagnostic value for the oocyte health status in E [20, 21].

Other mechanisms proposed to underly E that directly affect reproductive functions are alterations in microRNA (miRNA) expression levels [22]. MicroRNAs are a class of small regulatory non-coding RNA molecules (average 22 nucleotides in length) with high stability and abundance in cells and body fluids [23]. They regulate gene expression at a post-transcriptional level through inhibition of mRNA translation and/or mRNA degradation, and therefore play key roles in multiple biological processes [24]. Altogether, this makes them strong assets as biomarkers in diagnosis and treatment of diseases such as E [25, 26]. Identifying the altered miRNAs in E patients has been the goal of different studies by using different easy-access samples: serum [27, 28], plasma [29, 30], saliva [31, 32], as well as endometrial samples collected during surgeries [33, 34] and follicular fluid (FF) collected during oocyte retrieval [35–37]. Less attention has been paid to the impact E has on oocyte health itself and on associated CCs. So far, only miR-532-3p has been reported as altered in pools of CCs of the same patients diagnosed with advanced E compared to control patients (tubal/male factor) [38].

To date, there are no reliable tests that indicate how E affects oocyte quality or that help doctors to choose the best oocyte for IVF treatment. While a high-quality egg is essential for a successful pregnancy for any women undergoing fertility treatment, this is indeed particularly challenging in women suffering from E. Therefore, the objective of this study was to determine the impact of E on matured oocytes by identifying altered miRNAs in CCs from E compared to control patients by RNA-sequencing. Additionally, the study aimed to unveil if there is a differential miRNA footprint in CCs of oocytes that can be fertilized compared to the ones that fail fertilization in E patients. For this purpose, RNA-sequencing was performed on a CCs biopsy of one single cumulus-oocyte complex (COC) of one patient, without pooling different CCs biopsies from different cumulus-oocyte complexes (COCs) from the same patient. This approach allowed to identify a panel of miRNAs that can be used as biomarkers to select an oocyte with the best quality despite the E disease and with higher potential to be fertilized. These findings will help to create a truly non-invasive diagnostic test to assess egg quality and may lead to better treatments for women with endometriosis-related infertility in the future.

## Methods

### Cumulus cells sample collection

#### Patients

CCs were collected from oocytes from 33 women undergoing routine oocyte retrieval procedure for ICSI between 2019 and 2023 at the University Hospital Zurich following written informed consent (ethics committee, BASEC-Nr. 2018 − 00797). From a total of 33 patients, 15 were diagnosed with E (2 patients donated 2 samples each; total 17 CCs E samples), and 18 with other infertility problems (control, C) (11 male or and 7 non-hormonal factors) (1 patient donated 2 samples; total CCs samples 19). Patients with E were selected for the study based on the clinical history, covering different grades of E pathology according to rASRM classification (revised American Society for Reproductive Medicine [39]), and different E phenotypes, mainly regarding the presence and absence of lesions in the ovaries. Overall, from 15 E patients, 8 patients had mild E (M, stage I-II) versus 9 with severe E (S, stage III-IV) (in each group, one patient donated two CCs samples). Besides, 7 patients had ovarian lesions (endometrioma) (O), while 10 did not presented ovarian lesions (N) (in each group, one patient donated two CCs samples). The average age of the patients donating cumulus cells was 35.2 for E and 36.7 years for C patients.

#### Controlled ovarian stimulation

All patients received controlled ovarian stimulation (COS) with gonadotrophins for up to 13 days to allow simultaneous growth of multiple ovarian follicles before being triggered for superovulation. Prior to stimulation, women received gestagen (10 mg/d) for 10 up to 28 days, beginning at the second cycle day, in the short or antagonist protocols, and a GnRH agonist (triptorelin, 0.1 mg/d) on cycle day 21 for the long protocol of COS. For COS, either the short or long GnRH-agonist protocol or GnRH antagonist protocol were used with either application of hMG or recombinant FSH. When at least three follicles with a diameter of ≥ 17 mm were observed during transvaginal ultrasound, final oocyte maturation was induced with either 6500 IE hCG or with the addition of a GnRH-agonist accompanied by about 1600 IE hCG within the GnRH-antagonist protocol. Ultrasound guided oocyte retrieval was performed about 35 h after administration of the hCG/GnRH-antagonist trigger.

#### Cumulus cells collection, oocyte handling and oocyte fertilization

All cumulus-oocyte complexes (COCs) were collected at oocyte retrieval. A biopsy of CCs was collected using 8.0 × 0.30 mm insulin syringes. The CCs biopsy transferred to 0.2-µL tubes and immediately snap frozen in liquid nitrogen and stored at -80 °C. COCs were cultured in fertilization media (Global for Fertilisation, CooperSurgical, Trumbull, USA or G-IVF Vitrolife, Gothenburg, Sweden) under oil overlay (OVOIL, Vitrolife, Gothenburg, Sweden) in a humidified incubator at 37 °C, 20% O_2_ and 6% CO_2_. Two hours after retrieval, oocytes undergoing ICSI were denuded using hyaluronidase enzyme (80 IU/mL, HYASE-10X from Vitrolife, Gothenburg, Sweden). Mature oocytes (after visualization of the polar body prior to fertilization by ICSI) were collected and fertilized by ICSI while denuded immature oocytes were discarded. Following insemination incubated in Global Total or G1 Vitrolife media in microdroplet dish (Vitrolife) in MINC Benchtop incubator (COOK). Sixteen to 19 h after insemination oocytes were inspected for the presence of two pronuclei and two polar bodies (2PN). The 2PN zygotes selected for immediate day 2/3 or day 5 embryo transfer were left in culture. For some patients surplus 2PN zygotes were subjected to vitrification for future thawing cycles while for other patients, embryos were vitrified at blastocyst stage (day 5/6). Unfertilized oocytes, with absence of pronuclei (0PN) were discarded.

#### Retrospective classification of CCs

**Fig. 1:**
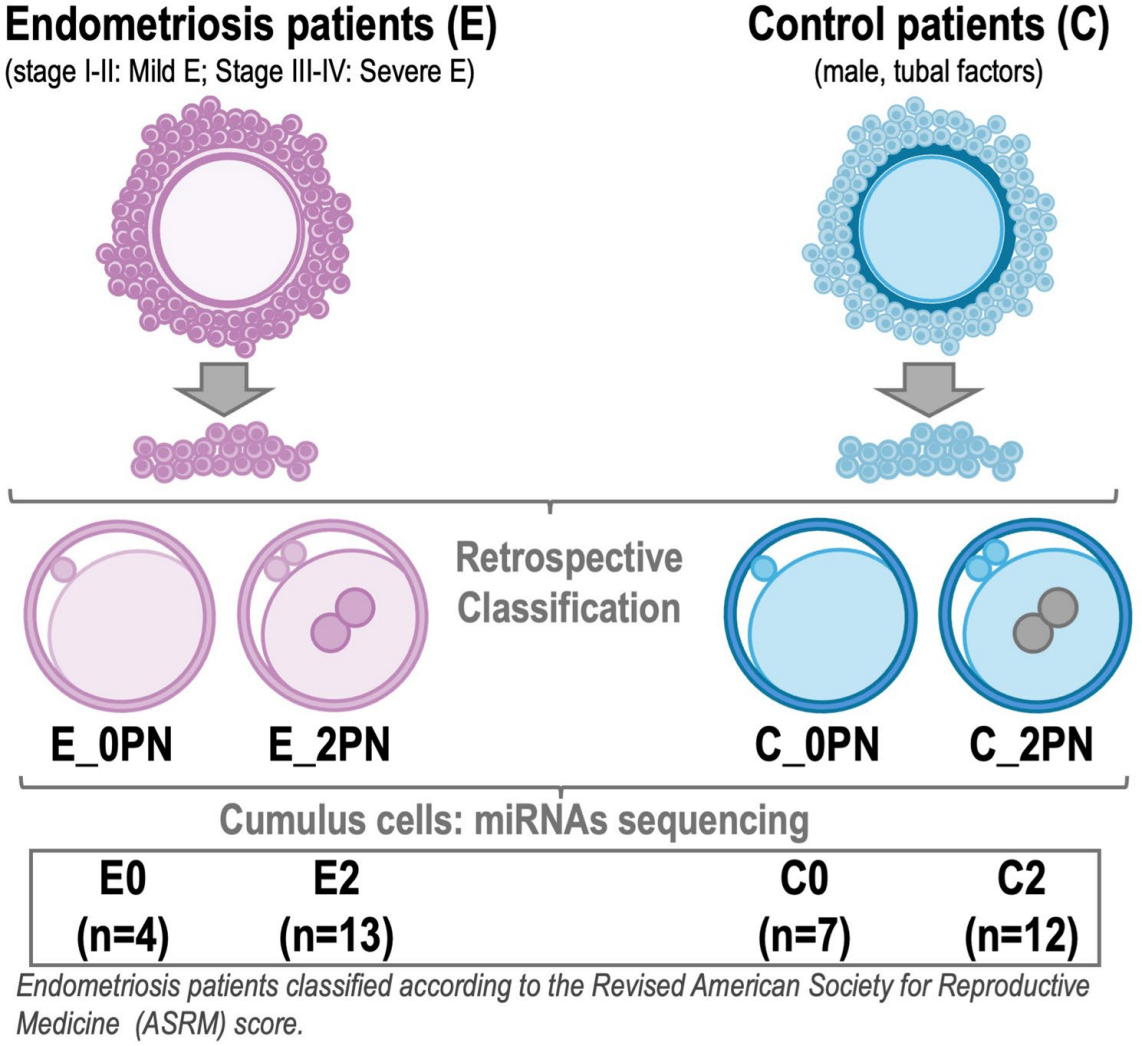
Schematic representation of the experimental design. Cumulus cells (CCs) from oocytes from endometriosis-associated infertility (**E**) and control (**C**) patients were collected and retrospectively classified in CCs from fertilized oocytes (2PN) and oocytes that failed fertilization (0PN). This resulted in four experimental groups: E2: CCs from fertilized oocytes in E; E0: CCs from non-fertilized oocytes in E; C2: CCs from fertilized oocytes in controls; and C0: CCs from non-fertilized oocytes in controls

To be able to analyze the miRNA signature of CCs from oocytes (MII) from E and C patients, which were successfully fertilized or not, a retrospective classification of the frozen CCs associated to mature oocytes was performed for all patients. The 4 experimental groups were: (1) E patients with fertilized oocytes (2PN) (group E2); (2) E patients with oocytes that failed fertilization (0PN) (group E0); (3) C patients with fertilized oocytes (2PN) (group C2); and (4) C patients with oocytes that failed fertilization (0PN) (group C0). From the 17 CCs of E patients, 13 CCs were classified as 2PN and 4 CCs as 0PN. From the 19 CCs samples of C patients, 12 CCs were classified as 2PN and 7 CCs as 0PN. A schematic representation of the experimental design and number of samples/experimental group is shown in Fig. 1.

### Isolation of RNA and small RNA-sequencing

#### RNA isolation from CCs

Total RNA from CCs samples was isolated using QIAzol lysis reagent (QIAGEN AG, Hombrechtikon, Switzerland) followed by the miRNeasy micro Kit (QIAGEN) protocol according to the manufacturer´s instructions. QIAzol lysis reagent was added to CCs samples while they were still completely frozen, followed by 2 min vortexing to allow complete sample disruption and homogenization. Concentration and RNA profiles were determined using Agilent 2100 Bioanalyzer RNA 6000 Nano and Pico assays (Agilent Technologies Schweiz AG). Besides, Agilent High Sensitivity RNA ScreenTape^®^ (Agilent TapeStation, Agilent Technologies Schweiz AG, Switzerland) was also used to measure RNA concentration and to obtain electrophoresis profiles of RNA samples. CCs samples with an RNA Integrity Number (RIN) [40, 41] of at least 6 were selected for preparation of small RNA-seq libraries (RIN range for E samples: 6.1–9.5 and RNA concentration 0.6–6.9 ng/µl; RIN range for C samples: 6.2–9.4 and RNA concentration 1.5–22.5 ng/µl). In total, 36 libraries were prepared: 17 libraries for E patients (13 libraries: E2 and 4 libraries: E0) and 19 libraries for C patients (12 libraries: C2 and 7 libraries: C0).

#### RNA library preparation and sequencing

RNA-Seq library preparation and sequencing were performed at the Functional Genomics Center Zurich (FGCZ; https://fgcz.ch). Library preparation started from approximately 1 ng of total RNA in 3 µl by using RealSeq^®^-AC miRNA Library Kit for Illumina^®^ sequencing (cat. no. 500 − 00048, BioCat GmbH, Heidelberg, Germany) following manufacturer´s instructions and with adaptor dilution 1:10. Sequencing of the obtained libraries was conducted on an Illumina NovaSeq 6000 instrument. Pooled barcoded libraries were run on one lane of a 10 B flow cell PE150.

#### RNA-seq data analysis

RNA-seq data was processed and analyzed using the public Galaxy Europe server version 24.1.4.dev. (https://usegalaxy.eu/) [42]. Fastq files were processed using fastp (fast all-in-one preprocessing for FASTQ files, Galaxy version 0.24.0 + galaxy3) to cut the read to 40 bases from the 3’-end, remove low quality bases at 3’-end (cut by quality in tail (3’) 1, cutting window size 5, cutting mean quality 30), clip adaptor sequences (TGGAATTCTCGGGTGCCAAGG), and discard reads shorter than 15 bases. Collapse sequences (Galaxy Version 1.0.1 + galaxy2) was used to get unique sequences and corresponding counts for each sample. MicroRNA reads were quantified with MiRDeep2 Quantifier fast quantitation of reads mapping to known miRBase precursors (Galaxy Version 2.0.0) based on human mature and precursor miRNA sequences from miRbase (mirbase.org). Differential expression analysis was performed using the Bioconductor package EdgeR (https://bioconductor.org/packages/edgeR/) [43].

### Validation of selected MiRNAs by quantitative real-time RT-PCR

Analysis of miRNA expression levels for two selected miRNAs based on RNA-seq results was performed in the remaining CC samples (24 samples) by quantitative real-time RT-PCR. The miRNAs analyzed were: hsa-miR-143-3p and hsa-miR-10b-5p. Taqman miRNA assays for each selected miRNAs (#A25576, ThermoFisher Scientific, Life Technologies Europe BV, Zug, Switzerland) are listed in Table 1. First, Taqman Advanced miRNA cDNA synthesis kit (#A28007, ThermoFisher Scientific) was used to generate cDNA from total RNA of CCs samples. The same RNA samples used for RNA-seq were used (2 µl total RNA input). Subsequently, miRNA expression levels of the selected miRNAs was examined in the obtained cDNA samples by real time PCR on a LightCycler 96 (Roche Diagnostics (Schweiz) AG, Rotkreuz, Switzerland) with TaqMan Fast Advanced Master Mix (#4444556, Thermo Fisher Scientific). The real-time PCR reactions were performed in 96-well plates at a final volume of 20 µL for all samples. Cycle parameters of the PCR were 1 cycle of enzyme activation at 95 °C for 20 s, followed by 50 cycles of denaturation at 95 °C for 3 s, and anneal/extension at 60 °C for 30 s. A non-template control (RNA sample) was included for each primer pair. The Cq values (quantification cycle) determined for the selected miRNAs were normalized against the geometric mean of two reference miRNAs (miR-191 and miR-200a). Differential expression between experimental groups in CCs samples were calculated and represented as 20-∆Cq. Statistical analysis was performed by using GraphPad Prisma software, version 8.2.0 (GraphPad Software, San Diego, CA, USA) (https://www.graphpad.com/scientific-software/prism/). Welch ‘s test unpair test was performed after examining data for normality. *P*-values < 0.05 were considered significant.

**Table 1:** MicroRNA (miRNA) sequences and corresponding assays used for validation experiments by quantitative real-time RT-PCR

miRNAs	Assay Reference ^a^	Sequence	Sequence length	Accession
hsa-miR-10b-5p	478494_mir	UACCCUGUAGAACCGAAUUUGUG	23	MI0000267
hsa-miR-143-3p	477912_mir	UGAGAUGAAGCACUGUAGCUC	21	MI0000459
hsa-miR-191-5p (reference miRNA)	477952_mir	CAACGGAAUCCCAAAAGCAGCUG	23	MI0000465
hsa-miR-200a-3p (reference miRNA)	478490_mir	UAACACUGUCUGGUAACGAUGU	22	MI0000737

^a^All assays were purchased from: Life Technologies, Paisley, UK

### Data mining and bioinformatics analysis of MiRNAs

Venn diagrams were generated with Jvenn (http://jvenn.toulouse.inra.fr/app/example.html) [44] to represent overlaps of differentially expressed (DE) miRNA of comparisons between experimental groups or for comparing target genes from miRNAs predicted in the present study to other studies. To examine miRNA target genes, MIENTURNET (http://userver.bio.uniroma1.it/apps/mienturnet/) [45] was used. The miRNA-target enrichment analysis was based on miRTarbase results with FDR 0.4. To convert gene symbols into Gene IDs, Synigo tool (https://www.syngoportal.org/convert) [46] was used. To obtain information about overrepresented biological GO terms and pathways DAVID (Database for Annotation, Visualization and Integrated Discovery) [47, 48] was used. KEGG pathway database (https://www.genome.jp/kegg/pathway.html) [49, 50] was applied to represent interesting pathways and associated target genes.

## Results

### Identification of differentially expressed MicroRNAs (miRNAs)

From a total of 36 libraries prepared, RNA-seq analysis provided sequence data for 36 libraries, 17 for E CCs samples and 19 for C CCs samples. After quality controls, three C2 samples were omitted from subsequent analysis due to very low read counts compared to the rest of the samples.

Overall, a total of 2652 miRNAs with at least 1 count in at least 1 sample were detected and 682 miRNAs with at least 10 counts over all samples. For further statistical analysis, miRNAs were included when they had at least 10 counts per sample in at least around 70–75% of samples of at least one experimental group. Based on these filtering thresholds, 201 miRNAs were selected for analysis comprising all experimental groups. Similar filtering was performed for the following statistical comparisons: E vs. C (resulting in 189 miRNAs); 2PN vs. 0PN (182 miRNAs), E2 vs. E0 (184 miRNAs), and C2 vs. C0 (194 miRNAs). In the case of the E samples, filtering for comparisons between E disease stage S vs. M and E lesions O vs. N, resulted in 181 and 193 miRNAs, respectively. All lists of identified, filtered and differentially expressed (DE) miRNAs can be found in Additional file Table S1-S9.

#### Differential MiRNAs in CCs samples between E and C

**Fig. 2:**
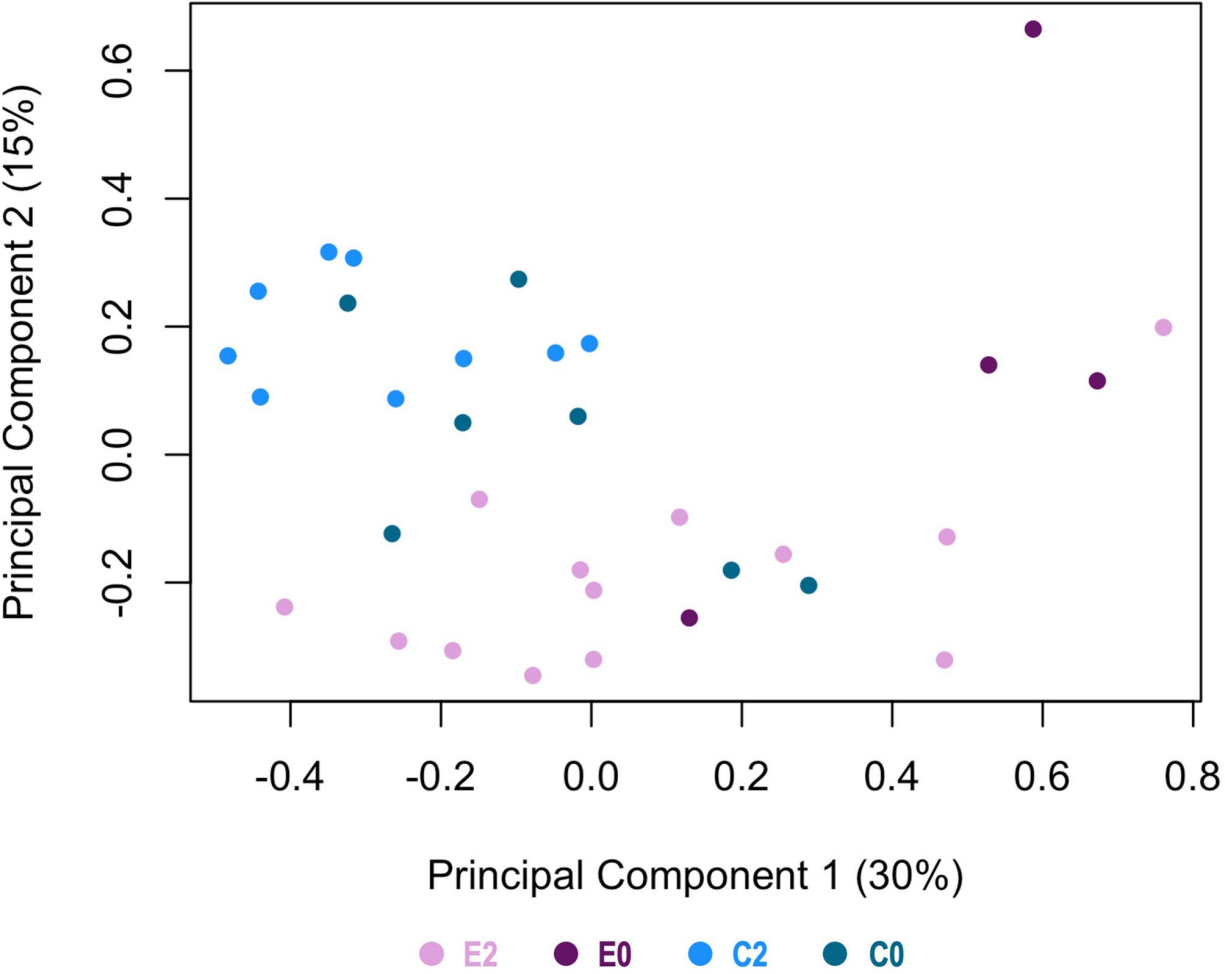
Principal Component analysis of all miRNAs identified in cumulus cells (CCs) for the four experimental groups. E2 in pink: CCs from fertilized oocytes in E; E0 in dark wine red: CCs from non-fertilized oocytes in E; C2 in light blue: CCs from fertilized oocytes in controls; and C0 in dark cyan: CCs from non-fertilized oocytes in controls

**Fig. 3:**
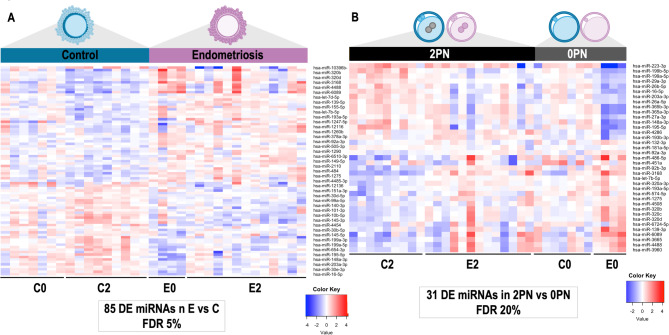
Comparative analysis of miRNAs identified in cumulus cells (CCs) from oocytes from endometriosis-associated infertility patients (E, including 2PN and 0PN), versus control patients (C, including 2PN and 0PN) regardless of fertilization and disease status and comparative analysis of miRNAs in CCs from fertilized (2PN, including E2 and C2) vs. non-fertilized (0PN, including E0 and C0) oocytes. (C: all control patients; E: all endometriosis patients; 2PN: all patients with fertilized oocytes; 0PN: all patients with oocytes that failed fertilization; E2: endometriosis patients with fertilized oocytes: E0: endometriosis patients with oocytes that failed fertilization; C2: control patients with fertilized oocyte; C0: control patients with oocytes that failed fertilization). **(A)** Heatmap representing differentially expressed (DE) miRNAs identified in E vs. C comparison derived from EdgeR analysis by Bioconductor package (False discovery rate, FDR < 0.05). **(B)** Heatmap representing DE miRNAs identified in 2PN vs. 0PN comparison (FDR < 0.20). Rows indicate single miRNAs while columns represent individual samples. Mean-centered expression values (log2 counts per million of sample – mean of log2 counts per million of all samples) for all samples are shown. Color scale in A is from − 4 (blue, lower than mean) to 4 (red, higher than mean) and in B from − 2 (blue, lower than mean) to 2 (red, higher than mean)

Principal component analysis (PCA) (Fig. 2) based on all 201 miRNAs revealed a grouping of C samples (including C2 and C0) and slight separation from the E samples (including E2 and E0) mainly in principal component 2. All C samples tended to group closer together while E samples were more spread in component 1, with E0 samples showing higher variability. For the E vs. C statistical comparison, 85 DE miRNAs (false discovery rate, FDR < 5%) were identified (59 DE miRNAs for FDR < 1%). In Fig. 3A, the heatmap represents the DE miRNAs between E and C samples, indicating a distinct molecular signature associated to CCs of E patients.

#### Differential MiRNAs in CCs samples between 2PN and 0PN oocytes

For the 2PN vs. 0PN statistical comparison, 37 DE miRNAs (FDR < 20%) were identified. In Fig. 3B, the heatmap represents the DE miRNAs showing significant differences in miRNAs profiles between 2PN (including E2 and C2) and 0PN (including E0 and C0) samples and indicating a distinct molecular signature associated with fertilized oocytes.

#### Differential MiRNAs in CCs samples between E2 and E0

**Fig. 4:**
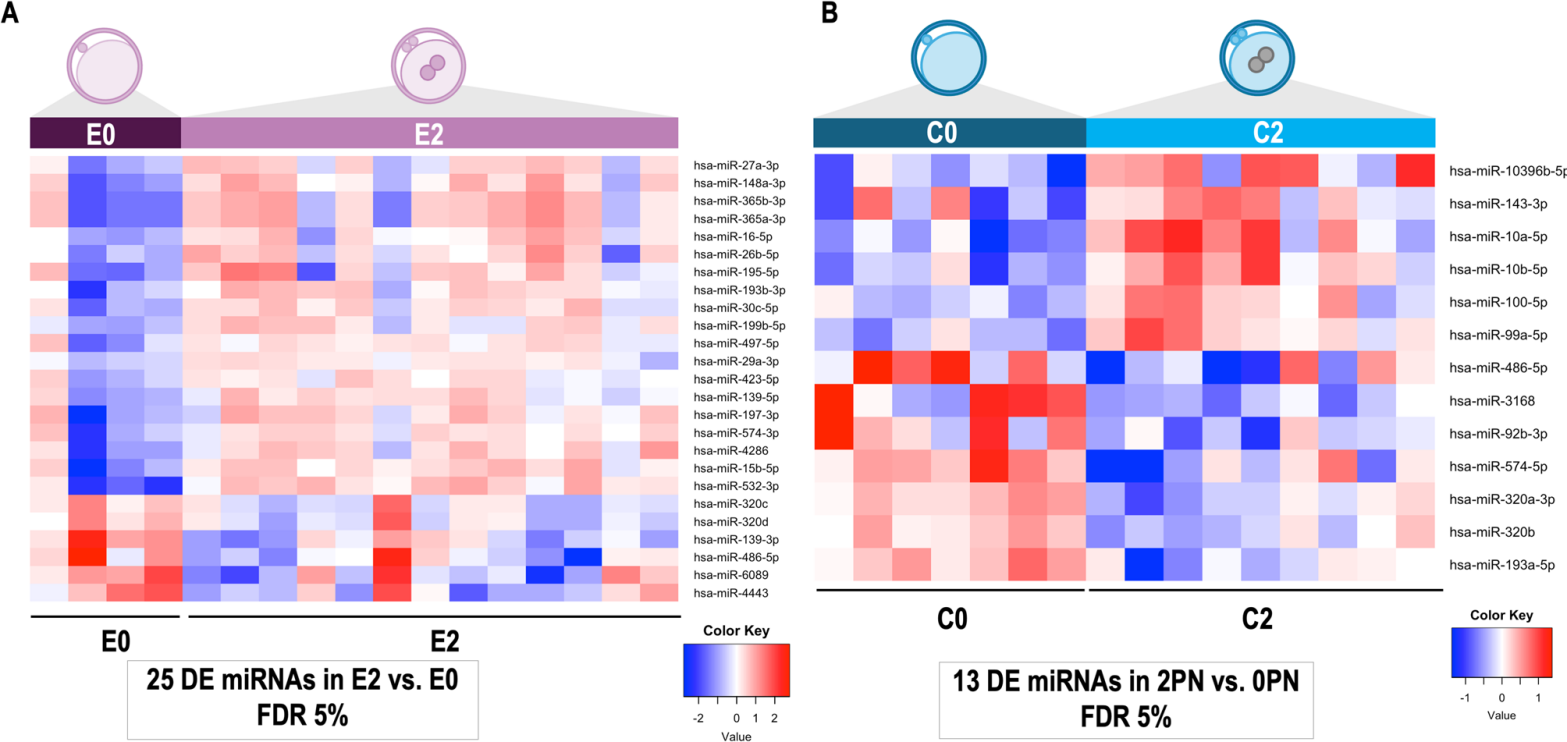
Comparative analysis of miRNAs identified in cumulus cells (CCs) from oocytes from endometriosis-associated infertility patients from fertilized (E2) versus non-fertilized oocytes (E0) and comparative analysis of miRNAs in CCs from control patients (C) from fertilized (C2) versus non-fertilized oocytes (C0). (**A**) Heatmap representing differentially expressed (DE) miRNAs identified in E2 vs. E0 comparison derived from EdgeR analysis by Bioconductor package (False discovery rate, FDR < 0.05). **(B)** Heatmap representing DE miRNAs identified in C2 vs.C0 comparison (FDR < 0.05). Rows indicate single miRNAs while columns represent individual samples. Mean-centered expression values (log2 counts per million of sample – mean of log2 counts per million of all samples) for all samples are shown. Color scale is A is from − 2 (blue, lower than mean) to 2 (red, higher than mean) and in B from − 1 (blue, lower than mean) to 1 (red, higher than mean)

Statistical comparison of E2 vs. E0 resulted in 25 DE miRNAs (FDR < 5%) (8 DE miRNAs for FDR < 1%). Significant differences in miRNA in E2 vs. E0 comparison are illustrated in the heatmap in Fig. 4A.

*Differential miRNAs in CCs samples from E patients with regard to severity of the disease and presence of ovarian lesions.* Statistical comparison of O vs. N resulted in 2 DE miRNAs (FDR < 20%), while for S vs. M no statistical differences were found.

#### Differential MiRNAs in CCs samples between C2 and C0 oocytes

Statistical comparison of C2 vs. C0 resulted in 13 DE miRNAs (FDR < 5%) (4 DE miRNAs for FDR < 1%). Significant differences in miRNA in C2 vs. C0 comparison are illustrated in the heatmap in Fig. 4B. The results of all comparisons can be found in Additional file Tables S4-S9.

### Comparison of DE MiRNAs identified in four of the group comparisons

**Fig. 5:**
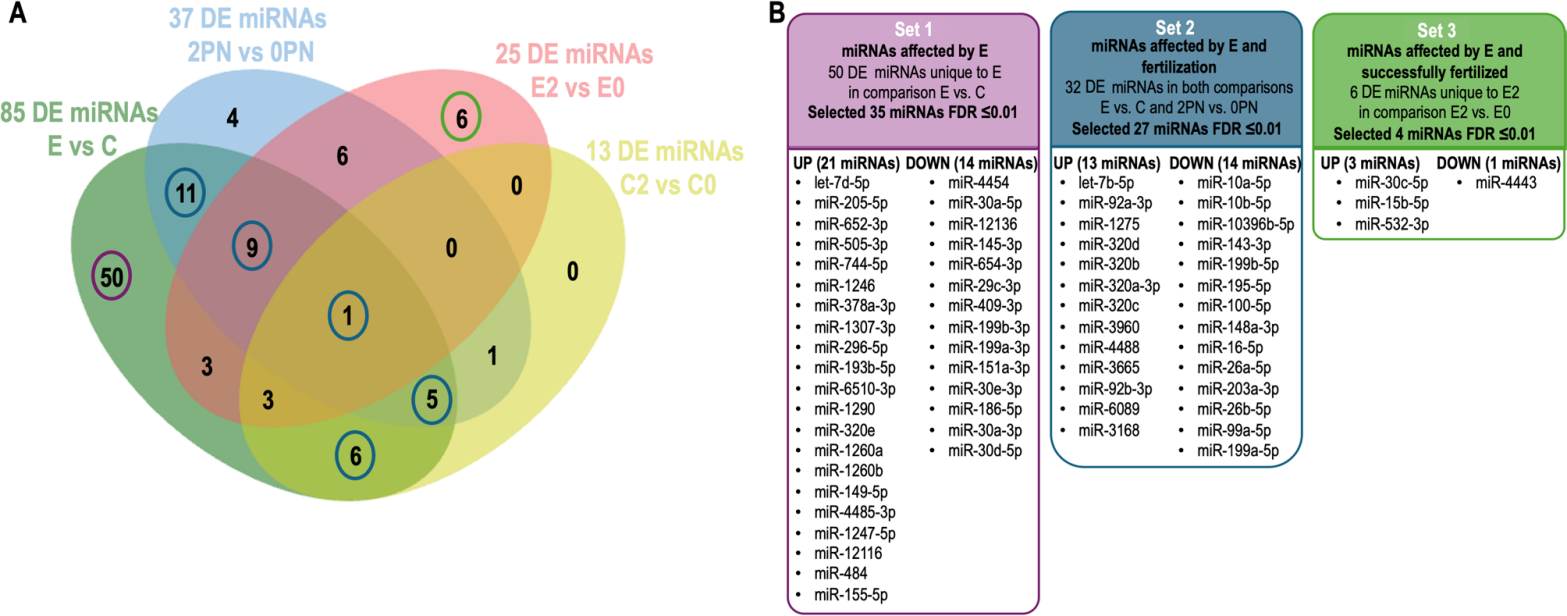
Comparison of miRNAs derived from the four statistical comparisons and selection of three sets of differentially expressed (DE) miRNAs: set 1, DE miRNAs affected only by endometriosis-associated infertility (E); set 2, DE miRNAs affected by E and the potential of the oocyte to be fertilized; and set 3, DE miRNAs affected by E but still with the potential to be successfully fertilized. (C: all control patients; E: all endometriosis patients; 2PN: all patients with fertilized oocytes; 0PN: all patients with oocytes that failed fertilization; E2: endometriosis patients with fertilized oocytes: E0: endometriosis patients with oocytes that failed fertilization; C2: control patients with fertilized oocyte; C0: control patients with oocytes that failed fertilization). **(A)** Venn Diagram showing overlaps of DE miRNAs of 4 statistical comparisons (E vs. C: 85 miRNAs; 2PN vs. 0PN: 37 miRNAs; E2 vs. E0: 25 miRNAs and C2 vs. C0: 13 miRNAs). (**B)** Illustration of the selected DE miRNAs (based on fold change FDR < 0.01) derived from the four different statistical comparisons in three sets of miRNAs: Set 1 oocytes uniquely affected by E; Set 2 oocytes affected by both E and the potential of the oocyte to be fertilized; and Set 3 oocytes affected by E but still fertilized despite the disease

To identify different sets of interesting miRNAs, the DE miRNAs obtained from the 4 comparisons E vs. C, 2PN vs. 0PN, E2 vs. E0, and C2 vs. C0 were compared in a Venn diagram. Figure 5A illustrates this comparison, displaying 50 miRNAs unique to E vs. C, 4 miRNAs unique to 2PN vs. 0PN, 6 miRNAs unique to E2 vs. E0, and no miRNA unique to C2 vs. C0. These comparisons also showed that 32 miRNAs were in common to E vs. C, and 2PN vs. 0PN or C2 vs. C0. Based on this comparison, three sets of miRNAs were selected (Fig. 5B): Set 1 with 50 miRNAs only significantly affected by E; Set 2 with 32 miRNAs affected by both E and the potential of the oocyte to be fertilized; and Set 3 with 6 miRNAs affected by E but still fertilized despite the disease. To be more restrictive for the subsequent functional enrichment analysis, DE miRNAs from the three sets with FDR < 1% were selected, resulting in 35 miRNAs in Set 1 (21 up- and 14 down-regulated in E vs. C), 27 miRNAs in Set 2 (13 up- and 14 down-regulated in E vs. C and with opposite regulation in 2PN vs. 0PN), and 4 miRNAs in Set 3 (3 up- and 1 down-regulated in E2 vs. E0). The complete list of selected miRNAs for the three sets can be found in Additional file Tables S10-S12.

### Functional enrichment analysis of potential target genes for selected DE MiRNAs

Mienturnet online tool identified predicted target genes for the selected DE miRNAs from the three sets: Set 1 (only affected by E, E vs. C), Set 2 (affected by E and the potential to be fertilized, common to E vs. C and 2PN vs. 0PN) and Set 3: affected by E and still being fertilized, E2 vs. E0) (FDR < 1%), separately for the miRNAs down- or up-regulated for each set. The number of potential target genes for the miRNAs in each set is summarized in Table 2 and complete lists of target genes can be found in Additional file Tables S13-S18. Next, functional enrichment analysis of predicted target genes for the three sets of DE miRNAs was performed using DAVID based on Gene Ontology (GO) Biological process, Molecular function, and cellular compartment as well as KEGG pathways, Reactome pathway and WIKI pathways. The terms with at least an enrichment score of 1.33 were considered as significant (this score corresponds to -log10 of *p*-value 0.05) and the resulting enriched functional terms can be found in Additional file Tables S19-S24.

**Table 2:** Number of predicted target genes for the three sets of MicroRNAs (miRNA) identified up and down regulated in cumulus cells

miRNA set	miRNAs up	Target genes	miRNAs down	Target genes
**1**	21	2831	14	2046
**2**	3	2973	14	2738
**3**	3	1402	1	108

Overall, the predicted target genes of miRNAs in the three sets, were overrepresented for GO terms and pathways related to cellular localization, regulation of gene expression, vesicle-mediated transport, extracellular vesicle (EVs), exosome, miRNAs targeting immune cells, chromatin and chromatin organization, and organelle organization (examples with the highest enrichment scores). Additionally, other interesting GO terms or pathways obtained as significantly overrepresented were oocyte maturation, embryo development, alteration mitochondria function, calcium signaling, ROS, alterations in spindle, microtubules and epigenetics modifications. These GO terms and pathways, which were previously found to be altered in E patients and/or related to oocyte quality [20, 21, 38, 51, 52], were selected and summarized in Table 3. In this Table 3, the number of predicted target genes for each set of miRNAs considering the up- and down-regulation is illustrated for each GO term or pathway. Moreover, for each GO term or pathway a significant enrichment score is represented (> 1.33, score corresponding to a *p*-value < 0.05).

**Table 3:** Summary of the gene ontology (GO) terms and pathways related to the predicted target genes (TG) of the differentially expressed (DE) MiRNAs in the three sets

GO TERMS AND PATHWAYS	SET 1 UP		SET 1 DOWN		SET 2 UP		SET 2 DOWN		SET 3 UP		SET 3 DOWN	
	NoTG	ES	No TG	ES	No TG	ES	No TG	ES	No TG	ES	No TG	ES
**Oocyte maturation**												
Progesterone-mediated oocyte maturation			19	3.2			29	2.8	18	3.1		
Oocyte meiosis			23	3.2					25	2.5		
Ovarian follicle development			13	3.2					8	1.8		
**Embryo development**												
Embryo development	220	5.7	170	5.7	225	4.3	200	3.7	117	5.6		
Embryonic pattern specification	18	1.9										
Embryonic stem cell pluripotency pathways			33	3.2	31	1.9	39	2.8	23	3.1		
**Mitochondria function**												
Mitochondrial translation	24	8.0			30	6.5	24	4.3				
Mitochondrion organization	83	3.3					85	5.7	35	1.4		
Mitochondrial transport	50	3.3			47	3.8	47	5.7	24	1.4		
Regulation of mitochondrial membrane permeability			11	2.2	17	2.2	14	2.3	8	1.4		
Autophagy of mitochondrion	19	3.1							12	3.8		
Apoptotic mitochondrial changes			13	2.2	17	2.2	15	2.3	9	1.4		
Mitochondrial ATP synthesis coupled electron transport							22	2.6				
Mitochondrial fatty acid oxidation disorders	7	2.5										
**Calcium Signaling**												
Calcium-mediated signaling									18	1.4		
Calcium ion transport							52	2.5				
Response to calcium ion			21	2.4								
Calcium-ion regulated exocytosis	13	2.0										
**Reactive oxygen species (ROS)**												
Response to oxidative stress	82	4.5	67	4.8	80	2.3	85	4.7	49	3.8		
Response to reactive oxygen species	42	4.5	42	4.8	38	2.3	49	4.7	30	3.8		
Response to hydrogen peroxide	25	4.5	25	4.8	24	2.3	26	4.7	18	3.8		
Regulation of oxidative stress-induced intrinsic apoptotic signaling	14	3.4	10	1.5								
**Spindle**,** microtubule**,** and extracellular matrix remodeling**												
Spindle organization	47	4.2	29	3.0	57	6.1	37	4.0	19	3.2		
Regulation of attachment of spindle microtubules to kinetochore	9	2.0			9	2.2			8	2.2		
Spindle checkpoint signaling	9	2.0					10	2.2				
Spindle microtubule					27	6.1	18	4.0	13	1.5		
Microtubule cytoskeleton organization	266	3.1	180	2.4	286	4.2	251	3.1	159	5.3		
Organelle transport along microtubule	17	2.0			20	2.1	23	1.9	14	2.3		
Chromatin organization	294	5.4	231	4.6	315	11	287	5.7	179	4.3	13	1.3
Sister chromatid segregation	37	4.2	28	3.0	44	6.1	35	4.0	21	3.2		
Chromosome organization	103	4.2	72	3.0	110	6.1	96	4.0	57	3.2		
Metaphase chromosome alignment	22	4.2			19	6.1	17	1.7				
Regulation of extracellular matrix organization	19	2.2					20	2.4	12	1.5		
Degradation of the extracellular matrix			27	1.9			33	1.7				
**Epigenetics modifications**												
Epigenetic regulation of gene expression			57	4.5	69	3.6						
Histone modifying activity	142	5.4	125	4.6	135	11	142	5.7	88	4.3		
Methylation	47	1.8			47	2.5						
Chromatin modifying enzymes			42	1.6							5	1.3

No TG: number of predicted target genes dysregulated by DE miRNAs identified in CCs for each set of up and down-regulated miRNAs; ES: enrichment score provided by DAVID functional annotation tool. The terms with at least an ES of 1.33 were considered as significant (this score is –log10 *p*-value 0.05)

For enriched GO terms associated to oocyte maturation (Table 3), the predicted target genes for Set 1 down-regulated and Set 3 up-regulated were involved in “progesterone-mediated oocyte maturation pathway”, “oocyte meiosis” and “ovarian follicle development”, while for target genes in Set 2 down-regulated were only related to “progesterone-mediated oocyte maturation pathway”. Particularly, the predicted target genes involved in “progesterone-mediated oocyte maturation pathway” and “oocyte meiosis” pathways were represented in respective KEGG molecular pathways in Figs. S1 and S2. In both pathways, the predicted genes targeted by miRNAs from Set 1, Set 2 or Set 3 down-regulated were illustrated as potentially up-regulated in red; and from Set 3 up-regulated as potentially down-regulated in blue. The predicted genes in common to several miRNAs from both sets are illustrated in grey. For enriched GO terms associated to embryo development (Table 3), a high number of target genes from the three sets regardless the regulation (except Set 3 downregulated) were associated to the term “embryo development” and with fewer number of target genes associated to “embryonic stem cells pluripotency pathways” The GO term “embryonic pattern specification” was only associated to target genes of Set 1 up-regulated miRNAs.

In the case of GO terms associated to mitochondria functionality (Table 3), the overrepresented terms with higher number of associated genes were found in Set 1 and Set 3 up-regulated and in Set 2 up- and down-regulated compared to the other sets (e.g., “mitochondrial translation”, “mitochondrion organization” and “mitochondrial transport”). Target genes of miRNAs in Set 1 and Set 3 up-regulated were also related to “Autophagy of mitochondrion”. Target genes of miRNAs only in Set 2 down-regulated were associated to “mitochondrial ATP synthesis coupled electron transport”, while target genes of miRNAs in Set 1 up-regulated miRNAs were related to “mitochondrial fatty acid oxidation disorders”.

Regarding calcium signaling (Table 3), a higher number of predicted target genes was identified for miRNAs in Set 2 down-regulated and related to “calcium ion transport”, followed by similar number of target genes in Set 3 up- and Set 1 down-regulated associated to “calcium-mediated signaling” and “response to calcium ion”. The term “calcium-ion regulated exocytosis” involved target genes for miRNAs in Set 1 up-regulated.

Regarding oxidative stress-related GO terms (Table 3), the higher number of predicted target genes from the three sets of miRNAs, regardless the regulation, were assigned to “response to oxidative stress”, followed by “response to oxygen species” and “response to hydrogen peroxide”. Only target genes of miRNAs in Set 1 were associated to “regulation of oxidative stress-induced intrinsic apoptotic signaling”.

Other biologically relevant enriched GO terms were related to abnormalities in spindle and microtubules and chromatin organization (Table 3), such as “spindle organization”, “microtubule cytoskeleton organization” and “chromosome organization”. These terms were similarly represented regarding the number of predicted target genes for Set 1 and Set 2 up- and down-regulated and Set 3 up-regulated miRNAs. By contrast, a smaller number of predicted target genes for miRNAs in Set 1, Set 2 and Set 3 up-regulated were related “regulation of attachment of spindle microtubules to kinetochore” while for target genes for miRNAs in Set 1 and Set 2 were associate to terms such as “spindle checkpoint signaling” and “metaphase chromosome alignment”. Additionally, a smaller number of predicted target genes were related to “regulation of extracellular matrix organization” and “degradation of the extracellular matrix” and only related to Set 1 and Set 2 down-regulated miRNAs.

Regarding epigenetic regulation (Table 3), GO terms such as “epigenetic regulation of gene expression”, “histone activity”, “methylation” and “chromatin modifying enzymes” were associated to target genes in Set 1, 2 and 3 miRNAs. Other interesting GO terms and pathways associated to hormone signaling were found with differences among the three sets, e.g.: “response to steroid hormone”, “steroid hormone receptor signaling”, “estrogen signaling pathway”, “androgen receptor signaling”, and “follicle stimulating hormone FSH signaling” (Additional file Tables S19-S24.

### MicroRNA validation by real-time RT-PCR

**Fig. 6:**
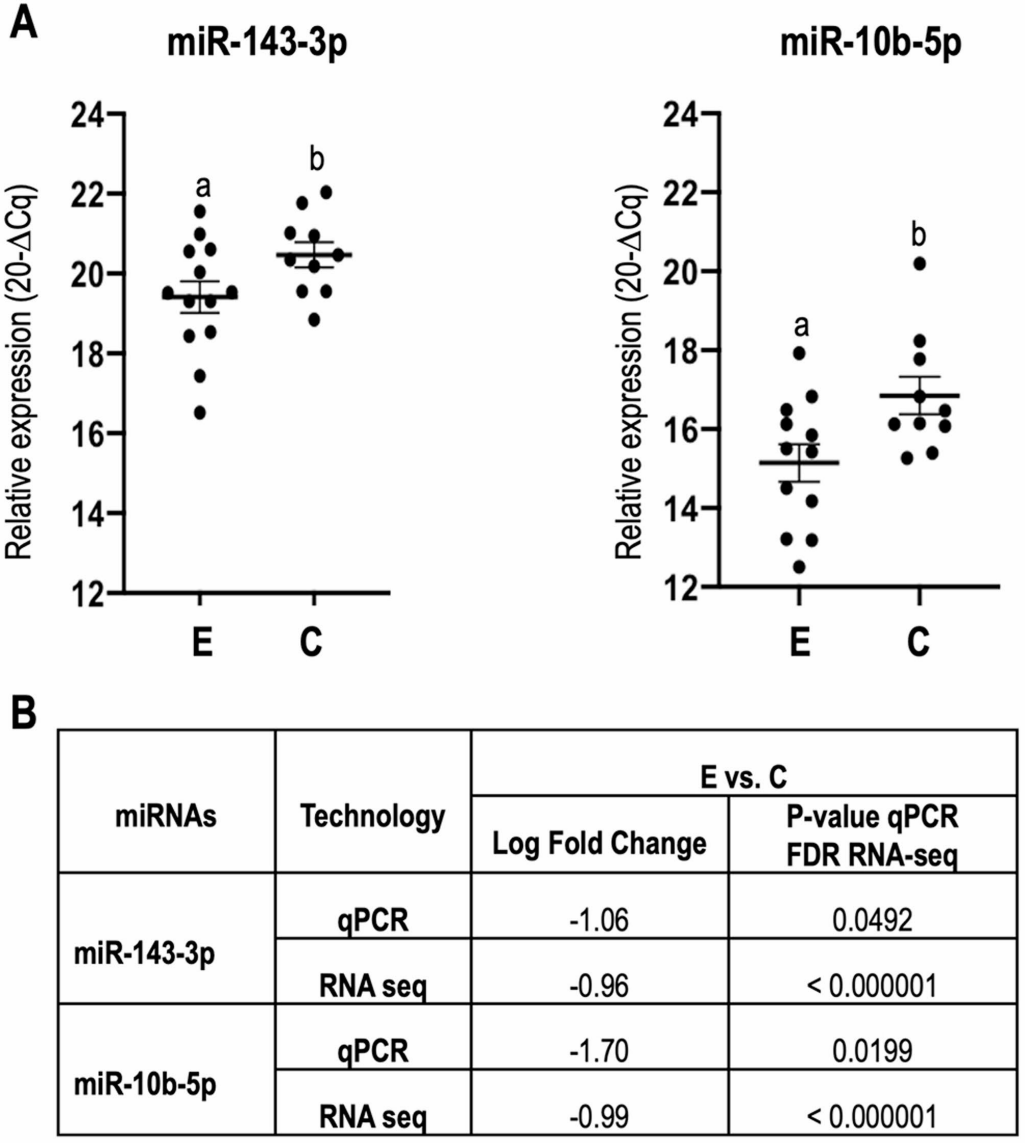
Validation of miRNAs by quantitative real-time RT-PCR (qPCR). (**A**) Graphs illustrate qPCR data of selected miRNAs, represented by the mean ± standard deviation of the mean (SEM) of the relative expression (20-∆Cq). **(B)** Comparison miRNA expression levels in cumulus cells (CCs) from oocytes by qPCR and RNA-sequencing (Fase discovery rate: FDR)

Validation of the RNA-seq results for two selected miRNAs, has-miR-143-3p and has-miR-10b-5p in 24 remaining CCs samples was performed by qRT-PCR. These miRNAs were selected because their target genes were involved in progesterone-mediated oocyte maturation and oocyte meiosis pathways. Results confirmed the RNA-seq data for the two selected miRNAs (Fig. 6).

## Discussion

The present study provides for the first time a clear differential miRNA signature in CCs from E patients compared to infertile C patients because of male factor or non-hormonal causes. The altered subset of CCs’ miRNAs identified in E patients have been associated with compromised oocyte competence, impaired oocyte maturation and their potential to be fertilized. Since it has been demonstrated that miRNAs in CCs can impact gene expression, meiotic progression, and developmental competence in the oocyte [53], the dysregulated miRNAs revealed here could be drivers of the impaired oocyte maturation and poor oocyte quality associated to E.

Our study identified 85 DE miRNAs (including miR-532-3p in Set 3), based on the analysis of biopsies of CCs from single oocytes analyzed by small RNA-sequencing. Our approach revealed individual variability, particularly among E patients, which could be due to the selection of E patients covering different grades of E pathology according to rASRM classification [39], and different E phenotypes/lesions [54]. Previously, da Silva et al. [38] analyzed CCs using a pool of undetermined number of CCs from the same patients with advanced E compared to C patients (tubal and male factor). This study reported one dysregulated miRNA (miR-532-3p) as a potential mechanism involved in the E etiopathogenesis, which also was identified in our study.

**Fig. 7:**
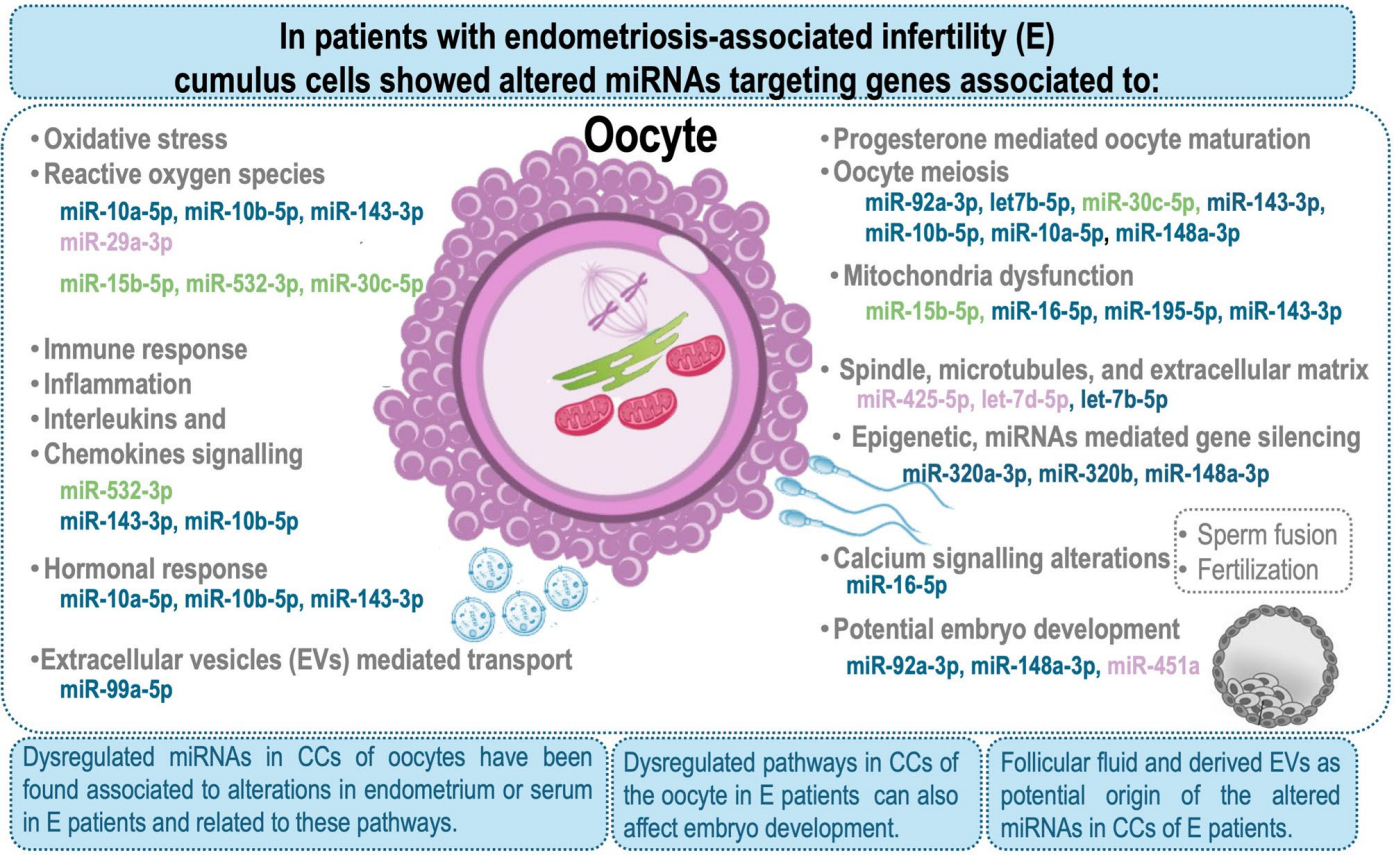
Diagram summarizing biological functions and pathways affected by endometriosis-associated infertility patients (E) and which genes could potentially be targeted by dysregulated miRNAs in CCs. MicroRNAs (miRNAs) highlighted in purple are displayed in Set 1 and refers to miRNAs only affected by E; in blue are miRNAs displayed in Set 2 and affected by both E and fertilization; and miRNAs in green are displayed in Set 3 and affected by E and successfully fertilized

The bioinformatics analysis of the data was mainly focused on selected DE miRNAs with the most significant differences (FDR < 1%) in three sets of miRNAs based on their association with E and/or potential of the oocyte to be fertilized. Comparison of these DE miRNAs with the current literature underlined that almost 80% of the DE miRNAs have been already associated to E (based on the analyses of endometrium, serum or EVs from liquid biopsies: FF, fallopian fluid, blood plasma, serum, peritoneal fluid; (Set 1: 26 out of 35, Set 2: 22 out of 27, Set 3: 4 out of 4). Functional overrepresentation analysis for the target genes of the DE miRNAs in the three sets predicted dysregulation of GO terms and pathways related to oocyte maturation, embryo development, alterations in mitochondria functionality, alterations in spindle and microtubules, ROS, calcium signaling, hormone response, immune response, and epigenetic regulation. Interestingly, most of the biological functions have been previously pointed as the major pathophysiological mechanisms affecting oocyte quality and embryo developmental competence in E patients [51]. The ROS production and secretion are significantly increased in E patients derived from high cell proliferation in ectopic endometriotic lesions, which present also compromised antioxidant enzyme functionality [51]. This increase in ROS and free radical production can lead to chronic inflammation, with increased secretion of interleukins (e.g., IL-6, IL-8, IL-12) and chemokines [51]. In this scenario, the overexpression of miR-15b-5p, miR-532-3p, and miR-30c-5p as found in E2 vs. E0 could play a protective role against oxidative stress as reported in different cell types [55–57]. In addition, miR-10b-5p, with decreased expression levels in E vs. C, targets different genes playing a role in a defense mechanism against reactive oxygen species (ROS) [58]. Similarly, decreased expression levels of miR-143-3p in E vs. C could contribute to overexpression of IL-6, HIF-1α and NF-κB p65, as observed in tumor growth [59]. Increased expression of HIF-1α is well-known in endometriotic lesions, particularly in ovarian endometriosis [60]. Moreover, alterations in HIF-1α expression in CCs during in vitro maturation impacts intercellular communication, steroid production, and, consequently, oocyte and blastocyst rates [61]. Regarding inflammation, downregulation of miR-10b-5p as in our study, resulted in increased IL-6 secretion, promoting inflammation in endometriosis [62]. Both ROS and inflammation can induce a detrimental effect on oocytes and the future embryo [51]. The predicted dysregulated GO terms and pathways in E patients derived from our analysis, together with a few examples of the identified altered miRNAs involved in each function have been summarized in Fig. 7.

We also found 6 miRNAs DE in CCs in our study, associated to poor oocyte quality or impaired oocyte competence. The three up-regulated miRNAs of Set 1, miR-320e, miR-505-3p, miR-1246, were also found as up-regulated in FF of women with poor quality oocytes [63]. The miRNAs miR-99a-5p, miR-10a-5p, and miR-10b-5p down-regulated in E vs. C (Set 2, affected by E and potential to fertilize) have been found as associated to poor oocyte competence in different species [64, 65]. In agreement with these findings in the literature, all three miRNAs showed lower expression in C0 vs. C2. In human, miR-99a-5p was identified in developmentally arrested oocytes versus normal oocytes [64]. In bovine, lower levels of miR-10a-5p have been associated to CCs of low competence oocytes [65]. In humans, miR-10a-5p was found as highly expressed in CCs of MII oocytes [66]. Additionally, the down-regulated miRNAs were found in FF or EVs-derived from FF in different species: miR-99a-5p in woman [67, 68] and miR-10b in woman [69, 70] and in bovine [65]. So far, all the mentioned miRNAs have been associated to E, except miR-505-3p. Altogether, this suggests that dysregulated expression levels of these miRNAs in CCs in E patients might lead to impaired oocyte competence and poor oocyte quality with a potential origin in FF surrounding the oocyte.

Regarding a role in oocyte maturation, several DE miRNAs were identified in the three sets of miRNAs. In the oocyte, miR-145-3p expression levels were lower in stage GV vs. MII stages [64], suggesting that the down-regulation of miR-145-3p in E vs. C in our study might reflect signs of incomplete oocyte maturation despite all selected oocytes showed nuclear maturation. In FF of polycystic ovary syndrome (PCOS) patients, miR-143-3p was identified up-regulated compared to healthy patients [71]. In contrast, miR-143-3p was down-regulated in E vs. C, which could be related to the opposite hormonal profile of both diseases [72]. Predicted target genes of miR-143-3p such as, *PTGS2*, *SERPINE1*, *CTGF*, *PGLS*, *TUBB2A*, *MIER3* have been found as altered in MII oocytes of E patients (both affected and non-affected ovaries) compared to control patients (healthy egg donors without endometrial pathologies) [73]. Particularly miR-143-3p, which was further validated by qPCR in our study, targets 5 genes involved in progesterone-mediated oocyte maturation pathway (*AKT1*, *AKT2*, *PIK3R1*, *MAPK1*, *IGFR1* and *CDC25B*), associated to key roles in different steps leading to a mature and competent oocyte.

Concerning progesterone-mediated oocyte maturation pathway and oocyte meiosis pathway, several miRNAs identified as dysregulated in our study are targeting specific genes involved in these pathways. Three miRNAs: miR-92a-3p, let7b-5p, and miR-30c-5p regulate *CDC25* expression while three others: miR-10b-5p, miR-10a-5p, and miR-148a-3p modulate *WEE1*. The encoded proteins of both *CDC25* and *WEE1* control meiotic resumption in the oocyte, modulate the activity of maturation promoting factor (MPF) [74], and are thus essential for oocyte maturation. In fact, oocyte maturation and its arrest at metaphase of meiosis II are regulated by changes in MPF activity. MPF consists of a complex of two proteins CDK1 and cyclin B1, which translation is also under the regulation of DE miRNAs found in our study. Cyclin B1 can be targeted by 4 miRNAs: miR-92a-3p, miR-92b-3p, miR-6089, and let7b-5p (Set 2 up-regulated) decreasing its expression levels, which will impact MPF activity. The mRNA of CDK1 could also be targeted by 4 miRNAs: miR-92a-3p (Set 2 up), miR-30c-5p (Set 3 up), miR-26-5p, and miR-143-3p (both Set 2 down), altogether inducing an imbalance of this protein, which would also affect MPF activity.

Other miRNAs have been related to alterations in oocyte maturation by impaired CCs expansion modulated by miR-378 (increased in E vs. C). Overexpression of miR-378 in CCs of porcine oocytes decreased expression of genes associated with CCs expansion (*HAS2*, *PTGS2*) and oocyte maturation (*GJA1*, *ADAMTS1*, *PGR*) [53]. Additionally, increased expression of miR-378 in CCs also suppressed oocyte progression from the GV to MII stage, accompanied by fewer oocytes from COCs overexpressing miR-378 reaching the blastocyst stage [53]. Knockdown of miR-378 led to increased CCs expansion and oocyte progression to MII, confirming a specific effect of miR-378 in suppressing COC maturation [53]. These results indicate that the miRNAs present in the CCs can impact oocyte maturation, while also implying that the manipulation of miRNA levels in CCs could influence oocyte in vitro maturation, pointing to therapeutical applications in ART.

Dysregulated miRNAs identified in E patients in our study were also associated to mitochondria dysfunction. Decreased mitochondria mass and abnormal mitochondrial structure have been reported in oocytes and CCs of E patients and associated to decreased oocyte quality [75, 76]. However, so far little is known about the role of the miRNAs in this matter. In our study, DE miRNAs in E patients have been identified as “mitochondrial-located miRNAs”, named mitomiRs, which impact mitochondrial function through the regulation of signaling pathways and mitochondrial-related proteins [77]. For example, miR-15b-5p, miR-16-5p, and miR-195-5p have been related to ATP production, miR-378a-3p to mitochondrial oxidative metabolism [78], miR-143-3p and miR-26a-5p to mitochondrial dysfunction, and let-7b-5p to mitochondrial-mediated ROS production [79]. Altogether, this suggests that dysregulation of mitomiRs could be associated to mitochondria of poorer quality in oocytes in E patients.

Regarding fertilization, several DE miRNAs identified in 2PN vs. 0PN have been associated to prediction of fertilization potential [68, 69, 80], e.g., miR-486-5p, miR-16-5p, miR-29a-3p, and miR-365a-3p. All of them, except for miR-365a-3p, were also DE for E vs. C. The 4 miRNAs target a set of 20 genes related to fertilization based on DAVID enrichment analysis. For example, miR-16-5p can target *HYAL3*, coding for a protein playing a key role in dispersing cumulus-oocyte complex layer and in penetrating the zona pellucida [81]. It also can target *ITPR1*, regulating fertilization and oocyte activation by tuning calcium oscillations [82].

Moreover, DE miRNAs identified in our study could also play a role during the oocyte-to-embryo transition. These miRNAs are referred as maternally inherited miRNAs, escaping the clearance of maternal transcripts during human oocyte-to-embryo transition to ensure their availability in later embryonic stages and contribute to normal embryo development [83]. Among them, miR-148a and miR-92a-3p, are examples of miRNAs that could impact early embryo development [84]. Another imbalanced miRNA decreased in E vs. C that could affect embryo development is miR-451a. Decreased expression of miR-451a was also detected in FF of E patients [35]. By knockdown miR-451, a decreased proportion of mouse/human MII oocytes developed into 2PN, 2-cell, 8–10-cell, and blastocyst stage embryos [35]. These findings point to miR-451a as a biomarker of oocyte quality and as a potential therapeutic target for E patients under ART.

Our study revealed for the first time an interesting profile of DE miRNAs from E2 vs. E0 comparison associated with a competent oocyte with the potential to achieve successful fertilization despite the E disease, while showing that E seems to have a more pronounced effect in oocytes that failed fertilization. The DE miRNAs with most significant differences in E2 vs. E0 represented in Set 3 have been previously related to E. The three miRNAs up-regulated in E2 vs. E0 (miR-532-3p, miR-15b-5p, and miR-30c-5p) have been reported as a part of the human MII oocyte signature [85], implying a competent oocyte profile. Among them, miR-30c-5p was found among the most significant miRNAs up-regulated in spent culture media of blastocysts from women with positive pregnancy [86] and women with successful implantation [87]. These findings bring valuable molecular clues to the existing literature. To date contradictory information can be found for the effect of E on fertilization rates and other reproductive outcomes [88–90], with the most recent reviews reporting no effect of E on fertilization rates [91].

The study might have been limited by the different distribution of the samples regarding the stage of the disease and the presence or absence of ovarian lesions in E2 and E0 (see Table S25 in Additional information) and the low number of samples in E0 experimental group. A follow-up study with a larger number of samples and a more homogeneous distribution of the samples per stage and lesion, will be necessary to elucidate if the differences reported for E0 vs. E2 are due to a direct and severe ovarian involvement in severe E0 patients. Current statistical comparisons in our study for O vs. N, showed only two differentially expressed miRNAs (FDR < 20%), with miR-139-3p associated to E [92], and no statistical differences for S vs. M.

The study might also be affected by the impact of the ovarian stimulation protocols and their individual adaptations, as a part of the ART treatment to increase the changes of retrieving a higher number of mature oocytes. On one hand, ovarian stimulation could potentially affect endometriosis lesions, progression of the disease or alter the ovarian hormonal environment. But to date there is only few reliable evidence for this and it is still under debate [93]. Nevertheless, the potential effects of the hormonal stimulation, such as a rise in peripheral estrogen concentrations are reached only for a few days before oocyte retrieval. Moreover, this occurs in both experimental groups E and C, since both were subjected to hormonal stimulation protocols. On the other hand, considering differential miRNAs identified in FFEVs from women treated with recombinant human chorionic gonadotropin (r-hCG) or with a gonadotropin-releasing hormone GnRH agonist (GnRH-a) for final oocyte maturation [67], similar numbers of patients for each ovarian stimulation protocol were selected in both experimental groups E and C to minimize this effect (see Table S25 in Additional information). Differential miRNAs in E vs. C identified in our study such as of miR-143-3p, miR-10b-5p, miR-10a-5p and miR-29a-3p have been related to imbalance steroid hormone synthesis in granulosa cells (estrogen, and estradiol) [94–97] and previously identidied as altered in endometriosis patients [30, 98–100]. Altogether, these differential miRNAs and others involved in main pathological pathways altered in endometriosis and discussed in the manuscript, reflect the different hormonal, ROS, and inflammatory milieu surrounding the oocyte in E, which can impact directly the oocyte or indirectly through alterations in CCs.

The results of this study provide a strong basis to develop personalized treatments to improve oocyte competence in ART based on miRNAs as therapeutic targets and inspire a novel non-invasive diagnosis of the impact of E on oocyte competence via CCs. To move towards this goal, further studies are needed to examine the mechanism how specific miRNAs, which have been identified here, contribute to oocyte competence and to understand the potential origin of dysregulated miRNAs in the follicular milieu. Despite the increasing interest in E research at clinical and molecular level, there is still a big gap in translating research results into new treatments and prevention. Since the impact of E on the oocyte itself and its potential to achieve a competent maturation and fertilization status have been less explored compared to other reproductive compartments, this translation at the clinical level seems even more far away. Bringing the molecular findings to the service of the E patients represents a valuable resource for selecting better oocytes leading to good embryos that can implant and thus lead to successful pregnancies.

## Conclusion

This study for the first time identified an altered miRNA signature in CCs of E patients, pointing towards compromised oocyte competence, impaired oocyte maturation and their potential to be fertilized. Besides, we revealed a CCs miRNA footprint in E patients for oocytes that can successfully be fertilized despite the disease. The study charts new territory for non-invasive diagnosis of the impact of E on oocyte competence using CCs and for personalized treatments improving oocyte competence in ART based on miRNAs as therapeutic targets.

## Supplementary Information

Below is the link to the electronic supplementary material.

Supplementary Material 1

Supplementary Material 2

Supplementary Material 3

Supplementary Material 4

Supplementary Material 5

## Data Availability

RNA-Seq data have been deposited at NCBI’s Sequence Read Archive (SRA), BioProject accession number PRJNA1238562 (https://www.ncbi.nlm.nih.gov/sra/PRJNA1238562). Further data supporting the findings of this study are available from the corresponding author upon reasonable request.

## Abbreviations

E: Endometriosis
CCs: Cumulus cells
COCs: Cumulus oocyte complexes
MicroRNAs: miRNAs
ART: Assisted reproduction treatment
DE: Differentially expressed
FF: Follicular fluid
rASRM: Revised american society for reproductive medicine
C: Control
E2: Endometriosis patients with fertilized oocytes
E0: Endometriosis patients with oocytes that fail fertilization
2PN: All fertilized oocytes (including endometriosis and control patients)
0PN: All oocytes that fail fertilization (including endometriosis and control patients)
EVs: Extracellular vesicles
