# Supplementary figures and images for: Endometriosis-associated infertility alters the microRNA signatures of cumulus cells with a particularly pronounced effect in oocytes that failed fertilization
Source: Biol Res. 2025 Sep 26;58:62. doi: 10.1186/s40659-025-00641-2 (PMC12465895; doi:10.1186/s40659-025-00641-2)

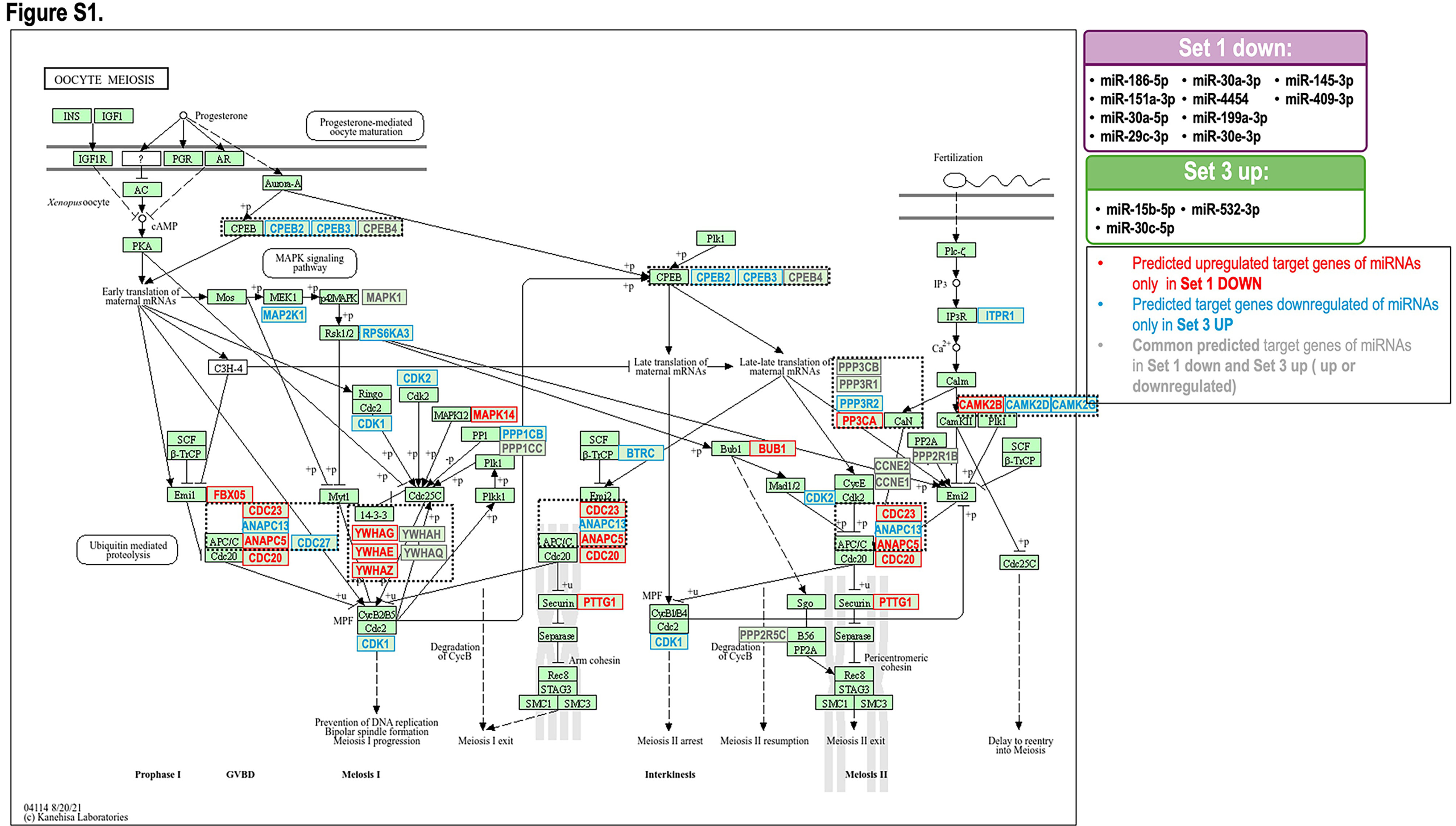

Supplement: Supplementary file 1 — Supplementary Material 1 [file 40659_2025_641_MOESM1_ESM.tif]

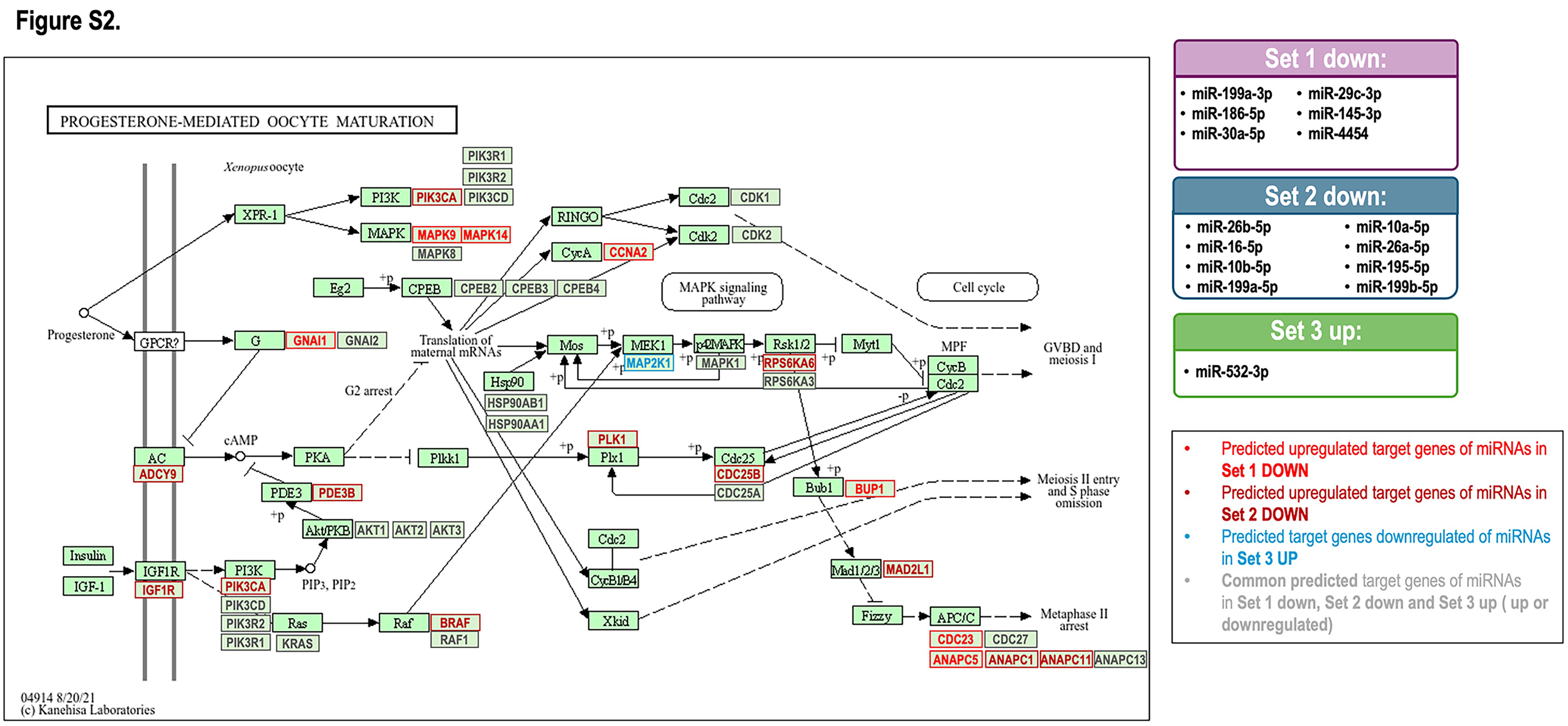

Supplement: Supplementary file 2 — Supplementary Material 2 [file 40659_2025_641_MOESM2_ESM.tif]
