# Supplementary material for: Endometriosis-associated infertility alters the microRNA signatures of cumulus cells with a particularly pronounced effect in oocytes that failed fertilization
Source: Biol Res. 2025 Sep 26;58:62. doi: 10.1186/s40659-025-00641-2 (PMC12465895; doi:10.1186/s40659-025-00641-2)
Supplement: Supplementary file 3 — Supplementary Material 3 [file 40659_2025_641_MOESM3_ESM.pdf]

KEGG Database Project
Kanehisa Laboratories
www.kanehisa.jp

Ref: 251395

Permission is granted toBiological Research to publish under the CC BY 4.0 open access license the following KEGG pathway map images in the article "Endometriosis-associated infertility alters microRNA signatures of cumulus cells with a particularly pronounced effect on oocytes that failed fertilization" written by Carmen Alminana and colleagues:

- Oocyte meiosis (hsa04114)
- Progesterone-mediated oocyte maturation (map04914)

subject to the condition that the original source is acknowledged by citing at least one KEGG paper.

Permission granted: Naomi Takahashi

Naomi Takahashi, Kanehisa Laboratories

Date: 23 May 2025

Copyright holder: Kanehisa Laboratories
