# Supplementary material for: Endometriosis-associated infertility alters the microRNA signatures of cumulus cells with a particularly pronounced effect in oocytes that failed fertilization
Source: Biol Res. 2025 Sep 26;58:62. doi: 10.1186/s40659-025-00641-2 (PMC12465895; doi:10.1186/s40659-025-00641-2)
Supplement: Supplementary file 4 — Supplementary Material 4 [file 40659_2025_641_MOESM4_ESM.pdf]

Kanton Zürich
Kantonale Ethikkommission

Prof. Dr. med. Erich W. Russi
Präsident Abteilung B

Dr. med. Peter Kleist
Geschäftsführer
Stampfenbachstrasse 121
Postfach
8090 Zürich
Telefon +41 43 259 79 70
Fax +41 43 259 79 72
admin.kek@kek.zh.ch
www.kek.zh.ch

Einschreiben
UniversitätsSpital Zürich
Reproduktions-Endokrinologie
Prof. Dr. med. Brigitte Leeners
Rämistrasse 100
8091 Zürich

30. Juli 2018 / ere

Beschlussmitteilung der Kantonalen Ethikkommission Zürich

Gesuch BASEC-Nr. 2018-00797
Biomarkers to Predict Successful Outcome of Fertility Treatments

Gesuchsteller Prof. Dr. med. Brigitte Leeners, USZ

Zentren Prof. Dr. med. Brigitte Leeners, USZ

I. Verfahren

☐ ordentliches Verfahren ☐ vereinfachtes Verfahren ☒ präsidiales Verfahren

II. Entscheid

☒ **Die Bewilligung wird erteilt**

Bedeutet: Das Vorhaben gemäss bewilligtem Forschungsplan kann gestartet und im Rahmen der anwendbaren rechtlichen Bestimmungen durchgeführt werden.

Bewilligungen für **klinische Versuche der Kategorie B und C** stehen unter dem **Vorbehalt**, dass

1. allfällig durch die zuständige eidgenössische Zulassungsbehörde (Swissmedic/BAG) festgestellte Mängel keine Änderungen der von der Ethikkommission evaluierten Unterlagen erfordern, und dass
2. die Bewilligung der eidgenössischen Zulassungsbehörde (Swissmedic/BAG) vorliegt.

☐ **Die Bewilligung wird mit Auflagen erteilt**

Bedeutet: Das Vorhaben gemäss bewilligtem Forschungsplan:

kann gestartet und im Rahmen der anwendbaren rechtlichen Bestimmungen durchgeführt werden.

Die Auflagen sind innert angemessener Frist zu erfüllen. Die revidierten Dokumente werden nach Einreichung im präsidialen Verfahren geprüft.

Folgende Auflagen müssen erfüllt werden:

Kontaktperson:

☐ **Gegenwärtig kann die Bewilligung noch nicht erteilt werden**

Bedeutet: Das Vorhaben kann **noch nicht** gestartet werden. Die nachfolgenden Bedingungen sind zu erfüllen. Die revidierten Dokumente werden nach Einreichung von der Ethikkommission geprüft.

Folgende Bedingungen müssen für alle Zentren erfüllt werden:

Kontaktperson:

☐ **Die Bewilligung wird nicht erteilt**

Bedeutet: Das Vorhaben kann in der vorliegenden Form nicht durchgeführt werden. Eine Neueinreichung ist möglich.

☐ **Auf das Gesuch wird nicht eingetreten**

Bedeutet: Die Ethikkommission ist für die Beurteilung rechtlich nicht zuständig (entweder ist eine andere Stelle für die Bewilligung zuständig, oder das Vorhaben kann ohne Bewilligung durchgeführt werden). Oder: Das Gesuch ist nicht vollständig.

☐ **Das Verfahren wird infolge Gegenstandslosigkeit abgeschrieben**
Bedeutet: Das Verfahren wird wegen Rückzugs des Gesuchs oder anderen Gründen gegenstandslos.

☐ **Das Verfahren wird sistiert**

☐ **Die Bewilligung wird entzogen**

III. Einteilung

☐ **Das Vorhaben gilt als klinischer Versuch gemäss KlinV**

- ☐ Kategorie ☐ A ☐ B ☐ C
- ☐ mit Arzneimitteln
- ☐ mit Medizinprodukten
- ☐ mit Transplantatprodukten
- ☐ der Gentherapie
- ☐ mit gentechnisch veränderten oder pathogenen Organismen
- ☐ der Transplantation
- ☐ anderer klinischer Versuch gemäss 4. Kapitel KlinV
- ☐ Umkategorisierung gemäss Art. 71 Abs. 3 KlinV, Kategorie ☐A ☐B ☐C
- ☐ mit Strahlenquellen

☒ **Das Vorhaben gilt als Forschungsprojekt gemäss HFV**

- ☐ Forschung mit Personen, Kategorie ☐A ☐B
- ☐ Umkategorisierung gemäss Art. 48 Abs. 2 HFV, Risiko-Kategorie ☐A ☐B
- ☐ mit Strahlenquellen
- ☒ Weiterverwendung biologischen Materials und/oder gesundheitsbezogener Personendaten
- ☐ Forschung mit verstorbenen Personen
- ☐ Forschung an Embryonen und Föten einschliesslich Totgeburten

☐ **Weiterverwendung ohne vorbestehende Einwilligung (Art. 34 HFG, Art. 37-40 HFV)**

a. Verwendungszweck
b. Bezeichnung des biologischen Materials/Personendaten
c. zur Weitergabe berechtigter Personenkreis
d. zur Entgegennahme berechtigter Personenkreis

☐ **Multizentrisches Forschungsprojekt**

☐BE ☐NZ ☐GE ☐OS ☐ TI ☐VD ☐ZH

Bitte beachten Sie, dass lediglich Dokumente, welche die Durchführung des Projektes am Hauptdurchführungsort und somit die Leit EK betreffen, geprüft werden. Es obliegt der Verantwortung des Gesuchstellers, sicherzustellen, dass Dokumente für beteiligte Zentren entsprechend angepasst und neu datiert werden.

IV. Begründung

Die Ethikkommission stützt ihre Begründung auf die Unterlagen, wie sie aufgeführt sind:

☒ in der submission summary vom 25.07.2018

☐ in der /den Stellungnahme/n der Kantonalen Ethikkommission/en:

☒ im Beschluss der Kantonalen Ethikkommission Zürich vom 11.05.2018

☐ sowie auf

☐ Wir bitten Sie, die geänderten Unterlagen im BASEC hochzuladen.

V. Kosten

Die Gebühren wurden bereits in Rechnung gestellt.

VI. Rechtsmittelbelehrung

Gegen diesen Beschluss kann innert 30 Tagen, von der Mitteilung an gerechnet, beim Regierungsrat des Kantons Zürich schriftlich Rekurs eingereicht werden. Die Rekursschrift muss einen Antrag und dessen Begründung enthalten. Der angefochtene Entscheid ist beizulegen oder genau zu bezeichnen. Die angerufenen Beweismittel sind genau zu bezeichnen und soweit möglich beizulegen.

VII. Mitteilung an den Gesuchsteller und in Kopie an:

☐ **Sponsor**

☐ **Swissmedic**

☐ **BAG**

☐ **beteiligte, lokale EKs (multizentrische Studien)**

☐ **Behörden:**

☐ **andere:**

VIII. Zusammensetzung der am Entscheid beteiligten Kommission

	Name, Vorname	Berufliche Stellung / Titel	m	f	am Beschluss beteiligt: ja	am Beschluss beteiligt: nein – abwesend	am Beschluss beteiligt: nein – in Ausstand
**Vorsitz**	Russi, Erich W.	Prof. Dr. med.	☒	☐	☐	☐	☐
**Mitglieder**	Ballmer, Peter E.	Prof. Dr. med.	☒	☐	☐	☐	☐
	Corti, Natascia	Oberärztin / Dr. med.	☐	☒	☐	☐	☐
	Eid, Karim	PD Dr. med.	☒	☐	☐	☐	☐
	Fetz, Daniel	Dr. phil. nat.	☒	☐	☐	☐	☐
	Grätz, Klaus W.	Prof. Dr. med., Dr. med. dent.	☒	☐	☒	☐	☐
	Hauser, Regula	Leiterin Weiterbildung, Institut für Hebammen/MPH	☐	☒	☐	☐	☐
	Hillinger, Sven	PD Dr. med.	☒	☐	☐	☐	☐
	Hoff, Paul	Prof. Dr. med. Dr. phil.	☒	☐	☐	☐	☐
	Iturrizaga, Raffael	Dr. phil.	☒	☐	☐	☐	☐
	Kobi Marius	Rechtsanwalt / lic. iur., LL.M.	☒	☐	☐	☐	☐
	Minder, Elisabeth	Prof. Dr.	☐	☒	☐	☐	☐
	Reinhart, Walter	Prof. Dr. med.	☒	☐	☐	☐	☐
	Schlüer, Anna-Barbara	PhD, MScN	☐	☒	☐	☐	☐
	Schönle, Eugen	Prof. Dr. med.	☒	☐	☐	☐	☐
	Schmid Büchi, Silvia	Dr. Pflegewissenschaft	☐	☒	☐	☐	☐
	Stahel, Rolf Arno	Prof. Dr. med.	☒	☐	☐	☐	☐
	Stocker, Hans*	Dr. phil. II	☐	☐	☐	☐	☐
	Weber, Konrad	PD Dr. med.	☒	☐	☐	☐	☐
	Weber, Markus	Prof. Dr. med.	☒	☐	☐	☐	☐

*für Biometrie zuständiges Mitglied

i.V. [signature]

Erich W. Russi

[signature]

Peter Kleist

Bemerkungen

Registrierungspflicht

Der Sponsor muss – falls es sich um einen klinischen Versuch handelt – diesen in einem WHO-Primärregister oder im Register der Nationalen Medizinbibliothek der USA (clinicaltrials.gov) erfassen und anschliessend diese Nummer im BASEC-Portal eingeben. Die Übertragung der erforderlichen Daten in das Swiss National Clinical Trials Portal (SNCTP) kann nach Bewilligung der Ethikkommission und Zustimmung des Gesuchstellers automatisch erfolgen. Die Informationen über den klinischen Versuch sind in beiden Registern öffentlich zugänglich. Zusätzlich veröffentlicht swissethics wenige Informationen wie Titel, Projekttyp oder Leit-Ethikkommission aller durch die kantonalen Ethikkommissionen bewilligten Gesuche auf swissethics.ch (ausser Phase-I-Studien).

Die Kantonale Ethikkommission Zürich bestätigt, dass sie nach ICH-GCP arbeitet.

Vorgehen zur Einreichung revidierter Dokumente

- Revidierte Unterlagen sind der Ethikkommission über BASEC zuzustellen.
- Die Änderungen sind in den revidierten Dokumenten zu markieren.
- Die revidierten Dokumente sind auch weiteren involvierten Zulassungsbehörden zuzustellen, sofern sie von diesen für die Bewilligung benötigt werden.

Meldungen und Berichterstattung an die Ethikkommission siehe Anhang 1 und Anhang 2

Anhang 1
Meldungen und Berichterstattung an die Ethikkommission ab 1. Januar 2014 für klinische Versuche (KlinV)

Meldung von Sicherheits- und Schutzmassnahmen
siehe Art. 37 KlinV:
Meldung an die EK innerhalb von 7 Tagen
Versuche mit Medizinprodukten: innerhalb von 2 Tagen

Abschluss, Abbruch oder Unterbruch des klinischen Versuchs
siehe Art. 38 KlinV:
Abschlussmeldung an die EK innerhalb von 90 Tagen
Abbruch- oder Unterbruchmeldung an die EK innerhalb von 15 Tagen
Schlussbericht an die EK: innerhalb 1 Jahres nach Abschluss/Abbruch

Schwerwiegende unerwünschte Ereignisse (Serious Adverse Events, SAE) bei klinischen Versuchen mit Arzneimitteln
Siehe Art. 40 KlinV:
Falls gemäss Protokoll nicht anders vorgesehen SAE mit Todesfolge innerhalb von 7 Tagen (an lokale EK nur lokale Ereignisse, an Leit-EK alle Ereignisse in der CH).

Verdacht auf eine unerwartete schwerwiegende Arzneimittelwirkung (Suspected Unexpected Serious Adverse Reaction, SUSAR)
Siehe Art. 41 KlinV:
SUSAR mit Todesfolge innerhalb von 7 Tagen, sonstige SUSARs innerhalb von 15 Tagen (an lokale EK nur lokale Ereignisse, an Leit-EK alle Ereignisse in der CH).

Schwerwiegende unerwünschte Ereignisse (Serious Adverse Events, SAE) bei klinischen Versuchen mit Medizinprodukten
Siehe Art. 42 KlinV:
Bei Versuchen der Kategorie C SAE bei Verdacht auf Zusammenhang mit Prüfprodukt oder erfolgtem Eingriff innerhalb von 7 Tagen (an lokale EK nur lokale Ereignisse, an Leit-EK alle Ereignisse in der CH).

Schwerwiegende unerwünschte Ereignisse (Serious Adverse Events, SAE) mit möglichem Zusammenhang zu untersuchter Intervention bei übrigen klinischen Versuchen
Siehe Art. 63 KlinV:
Meldung an EK innerhalb von 15 Tagen.

Berichterstattung über die Sicherheit der teilnehmenden Personen
Siehe Art. 43 KlinV:
1 mal jährlich Auflistung der Ereignisse weltweit (Annual Safety Report)

Mit dem jährlichen Sicherheitsbericht sind der EK auch alle Änderungen zu melden, die nicht bewilligungspflichtig sind (d.h. alle Änderungen, die gemäss Art. 29 KlinV nicht als wesentliche gelten).

Anhang 2
Meldungen und Berichterstattung an die Ethikkommission ab 1. Januar 2014 für Forschungsprojekte mit Ausnahme der klinischen Versuche (HFV)

Forschung mit Personen, die mit Massnahmen zur Entnahme biologischen Materials oder zur Erhebung gesundheitsbezogener Personendaten verbunden
Sicherheits- und Schutzmassnahmen siehe Art. 20 HFV
Meldung an die EK innerhalb von 7 Tagen

Schwerwiegende Ereignisse siehe Art. 21 HFV
Meldung innerhalb von 7 Tagen (an lokale EK nur lokale Ereignisse, an Leit-EK alle Ereignisse in der CH) und Unterbruch des Forschungsprojektes.

Abschluss und Abbruch des Forschungsprojekts siehe Art. 22 HFV
Meldung an die EK innerhalb von 90 Tagen

Weiterverwendung biologischen Materials und gesundheitsbezogener Personendaten für die Forschung
Siehe Art. 36 HFV:
Wechsel Projektleitung: Meldung an die EK: vorgängig

Abschluss und Abbruch des Forschungsprojekts
Meldung an die EK innerhalb von 90 Tagen

Weiterverwendung biologischen Materials und gesundheitsbezogener Personendaten für die Forschung bei fehlender Einwilligung und Information nach Artikel 34 HFG
Siehe Art. 40 HFV:
Änderungen der in der Bewilligung genannten Angaben
Meldung an die EK (vorgängig)

Abschluss oder Abbruch des Forschungsprojekts
Meldung an die EK innerhalb von 90 Tagen

Forschung an verstorbenen Personen (Art. 43 HFV)
Siehe Art. 43 HFV:
Wechsel der Projektleitung: Meldung an die EK (vorgängig)

Bei Forschungsprojekten mit verstorbenen Personen, die künstlich beatmet werden
Wesentliche Änderungen des Forschungsplans
Meldung an die EK (vorgängig)

Abschluss oder Abbruch des Forschungsprojekts
Meldung an die EK innerhalb von 90 Tagen

Submitted Documents 2018-00797

Submitted documents main centre

**Prof. Dr. med. Brigitte Leeners, Dept. of Reproductive Endocrinology, University Hospital Zurich, 8091 Zurich**		
File name	Version	Date of doc.
1. Cover Letter		
01a_Cover letter_2018_06_13.pdf		13/06/2018
Cover letter_2018_06_26.pdf		26/06/2018
2. Synopsis of the study plan		
no synopsis needed		
3. Participant information sheet and informed consent (ICF)		
Pat_Einwilligung_2018_06_11_clear.docx	V2.0	11/06/2018
Pat_Einwilligung_2018_06_11_highlighted.docx	V2.0	11/06/2018
4. Study plan (protocol), signed and dated		
02a_Study protocol_2018_06_26_clear.docx	V2.1	20/06/2018
02a_Study protocol_2018_06_26_highlighted.docx	V2.1	20/06/2018
Ethics-biomarker_Sign2018_06_20.jpeg	V2.1	20/06/2018
6. Investigator's CV, dated		
CV_Leeners_D_2018_05_01.doc		01/05/2018
14. Information on secure handling of biological material and personal data, and in particular on the storage thereof		
see doc/cat: **01**, page/ref: **Page 10**		
30. Proof of secure and correct coding		
see doc/cat: **01**, page/ref: **Page 11**		
39. Miscellaneous / Varia		
Biobank Reglement_2018_05_01_FV.docx	V 1.0	27/04/2018

25.07.18

Kantonale Ethikkommission Zürich
Stampfenbachstrasse 121
8090 Zürich
E. Red
